# Neglected Papillary Thyroid Carcinoma Seven Years after Initial Diagnosis

**DOI:** 10.1155/2013/148973

**Published:** 2013-01-14

**Authors:** Eleftherios D. Spartalis, Theodore Karatzas, Petros Charalampoudis, Chrysovalantis Vergadis, Dimitrios Dimitroulis

**Affiliations:** ^1^Second Department of Propedeutic Surgery, Laiko General Hospital, University of Athens, Medical School, 11527 Athens, Greece; ^2^Department of Radiology, Laiko General Hospital, 11527 Athens, Greece

## Abstract

Papillary thyroid carcinoma (PTC) is the most common epithelial thyroid tumor, accounting for more than 80% of all thyroid tumors. Recent advances in ultrasonographic screening and US-guided fine-needle aspiration biopsy (FNAB) have facilitated the early detection and diagnosis of papillary thyroid carcinomas. In exceptionally rare cases, papillary thyroid tumors may assume enormous dimensions due to recurrent disease or the patient's negligence of the problem. We report an extremely rare case of a 72-year-old woman presented with a neglected giant exophytic papillary thyroid carcinoma with hemorrhagic ulcers. Computed tomography showed a mass measured 17 × 12 cm that caused a displacement of the trachea to the right side and reached the mediastinum. After bleeding management, patient was discharged. The patient was fully aware of her situation, but she denied any further therapeutic management.

## 1. Introduction

It is well known that papillary thyroid carcinoma (PTC) has a generally indolent character and has a favorable prognosis if no high-risk features such as clinical lymph node metastasis, distant metastasis, and significant extrathyroid extension are present. Papillary thyroid carcinoma is a slow growing neoplasm which explains the relatively long duration until a diagnosis is established. Appropriate early surgical treatment decreases the risk of metastasis and recurrence. A neglected papillary thyroid carcinoma can occasionally assume large dimensions and present as a giant hemorrhagic goiter.

## 2. Case Presentation

**Figure 1:**
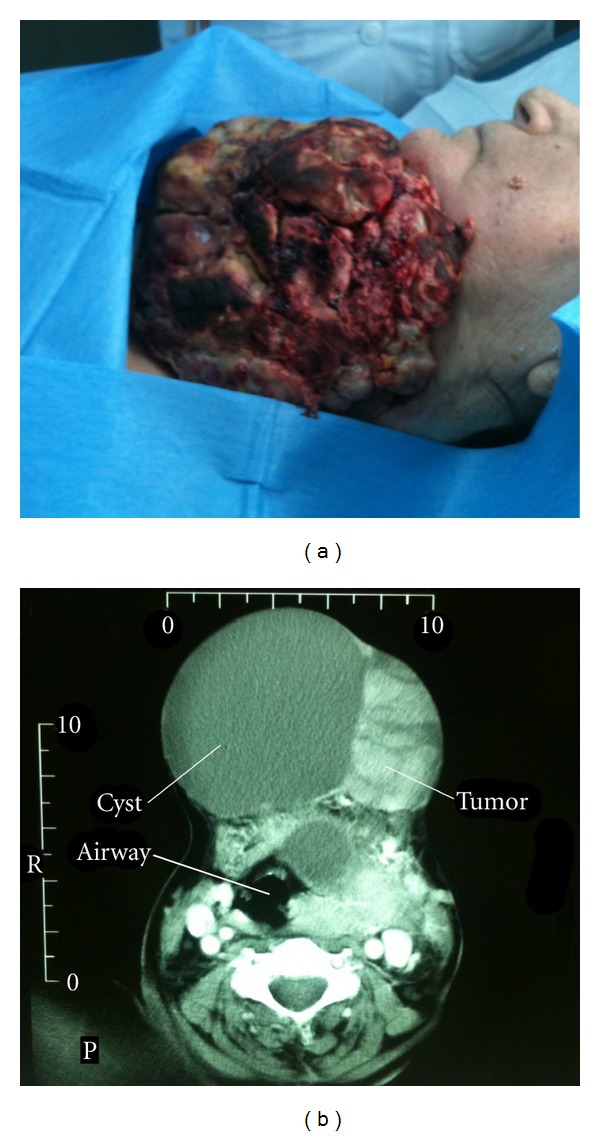
(a) Macroscopic appearance of the left hemorrhagic cauliflower-like side of the goiter. (b) Contrast-enhanced axial CT imaging shows a huge heterogenous mass localized to the left thyroid lobe and extending to the anterior neck space with a solid enhanced component and cystic areas. The trachea is displaced to the right side, and the mass reached the mediastinum.

A 72-year-old woman was admitted to our hospital with cervical bleeding due to a giant ulcerous goiter (Figure 1(a)). The patient had a long-standing history of neglected thyroid carcinoma. Seven years earlier, she had undergone excisional biopsy of the thyroid tumor, and histology revealed papillary carcinoma (PTC). After the diagnosis was established, the patient refused therapeutic treatment. The growing mass was regularly monitored by computed tomography (CT) over 7 years, although the patient continued to refuse therapy. A physical examination revealed a cauliflower-like cervical mass with a giant cystic section on the right side. The rest of the clinical investigation was unremarkable. Routine laboratory tests, including hematocrit, were within normal levels. The latest contrast-enhanced axial CT image showed a large heterogenous mass localized to the left thyroid lobe and extended to the anterior neck space with a solid enhanced component and cystic areas (Figure 1(b)). The neck mass at this time measured 17 × 12 cm and caused a displacement of the trachea to the right side. Mass surface bleeding was managed with common bedside hemostatic maneuvers and use of local electrocauterization. The patient was discharged after successful hemostasis, as she denied any further management, such as surgical debulking and radioiodine therapy. 

## 3. Discussion

In the differential diagnosis of a neck mass, neoplastic, congenital, traumatic, and inflammatory lesions and metabolic disorders should be considered [1]. The diagnosis is made by clinical and radiological evaluations and confirmed by biopsy and histologic evaluation. Fine needle aspiration biopsy (FNA-b) is an excellent tool for evaluating neoplastic neck masses [2].

Metabolic disease involving the thyroid and parathyroid glands can present as a neck mass. A goiter can present in an otherwise asymptomatic person. A variety of benign and malignant tumors such as lipoma, lymphoma, epidermoid carcinoma, thyroid tumors, melanoma, sarcoma, plasmacytoma, schwannoma, paraganglioma, and metastatic head and neck carcinoma may present as a neck mass [1, 2].

Congenital lesions are not always present at birth and can appear from birth to 30 years of age or older. Branchial cleft cysts, thyroglossal duct cysts, hemangiomas, and lymphangiomas are the most common congenital lesions that present as a neck mass. On the other hand, degenerative lesions are rare with laryngocele and Zenker's diverticulum being the most common causes [3].

Thyroid cancer is the most common endocrine malignancy. Papillary thyroid cancer (PTC) is the most frequent histological subtype, accounting for 80% of cases [4]. It occurs more often in the third and fourth decades. Over the last decades, the incidence of papillary thyroid carcinoma has increased. This apparent increase is due to the widespread use of cervical ultrasound and ultrasound-guided fine-needle aspiration biopsies of nonpalpable thyroid nodules and more accurate histopathologic screen for small PTMC with more extensive sampling of resected thyroids [5, 6]. Papillary thyroid carcinoma is associated with good outcome, and generally, the tumor is indolent with a low distant metastatic potential. The factors significantly associated with prognosis include male sex, aged over 45 years, local invasion, large tumor size, distant metastases, and histopathological tumor features [7, 8]. The prognosis is better in female patients and patients younger than 45 years [7]. The 5-year survival rate for local disease is 99.7%, whereas patients with regional metastases have a 96.9% 5-year survival rate. Patients with distant metastases have a 56% 5-year survival rate [6].

Despite its well-differentiated characteristics, PTC may be overtly or minimally invasive. Papillary tumors tend to invade lymphatics but are less likely to invade blood vessels. Of patients with PTC, about 11% present with metastases outside of the neck and mediastinum [8, 9]. Metastases, in descending order of frequency, are most common in neck lymph nodes and the lungs, followed by bones, the brain, the liver, and other sites [9].

Lymph node metastasis at the initial diagnosis is common, with rates as high as 78% [10]. Although common, it appears that the presence of positive lymph nodes has little influence on overall survival, although this does influence recurrence [10, 11].

In exceptionally rare cases, papillary thyroid tumors may assume enormous dimensions due to recurrent disease or the patient's negligence of the problem. If neglected, any thyroid cancer may exhibit symptoms due to compression and infiltration of the cancer mass into the neighboring tissues. After a thorough search of the English literature, only few other cases of a neglected papillary thyroid carcinoma with the enormous dimensions observed in this case have been reported to the best of our knowledge [12, 13]. There are also two articles regarding distant metastases from a neglected thyroid papillary carcinoma [14, 15].

Floros and Grigg reported a case of a 55-year-old woman with an enormous recurrent papillary thyroid carcinoma. She presented with two neck masses that measured 19 × 12 cm on the right and 17 × 9 cm on the left. The elevated serum TSH, history of papillary thyroid carcinoma, and radiological findings suggested the recurrence of papillary thyroid carcinoma. She underwent a two-stage debulking of the tumors and then received I-131 ablation therapy for residual disease [12]. Rush and Trinkle point out that even long-standing malignant lesions may be amenable to surgery. Operation is at times simplified by resultant displacement of normal anatomic structures [16].

In the second case, a 94-year-old woman with a large recurrent papillary thyroid carcinoma was treated with Mohs chemosurgery with good results. The tumor measured 10 cm and was exophytic and hemorrhaging. With Mohs chemosurgery, the tumor had flattened and the hemorrhage was stopped [13]. 

Mostarchid et al. reported a case of giant skull and brain metastasis from a neglected thyroid papillary carcinoma [14], while Pavlidis et al. investigated the occurrence of subcutaneous scalp metastases [15].

It is notable that the patient was fully aware of her situation, and there were no diagnosed mental health disorders that could inhibit understanding of the severity of the problem. The patient consulted several surgeons who advised that the tumor should be removed before growth and pressure to neighboring anatomical structures occurred.

## 4. Conclusion

Papillary thyroid cancer is well known for its low malignant potential and good prognosis. The outcome can be fatal in patients with low compliance and delayed treatment. The inability of health care access, lack of patient concern, and denial of the appropriate treatment could result in extreme cases of neglected papillary thyroid carcinoma, such as that presented here.
